# Thanks to all those who reviewed for *Systematic Reviews* in 2015

**DOI:** 10.1186/s13643-016-0205-9

**Published:** 2016-02-24

**Authors:** David Moher, Paul Shekelle, Lesley A. Stewart

**Affiliations:** Clinical Epidemiology Program, Ottawa Hospital Research Institute, School of Epidemiology, Public Health and Preventive Medicine, University of Ottawa, Ottawa, Canada; Canadian EQUATOR Centre, Ottawa, Canada; Ottawa Hospital, Ottawa, Canada; Division of General Internal Medicine, West Los Angeles VA Medical Center, Los Angeles, USA; Centre for Reviews and Dissemination, University of York, York, UK

## Abstract

A peer-reviewed journal would not survive without the generous time and insightful comments of the reviewers, whose efforts often go unrecognized. Although final decisions are always editorial, they are greatly facilitated by the deeper technical knowledge, scientific insights, understanding of social consequences, and passion that reviewers bring to our deliberations. For these reasons, the Editors-in-Chief and staff of *Systematic Reviews* warmly thank the 282 reviewers whose comments helped to shape the journal, for their invaluable assistance with review of manuscripts in Volume 4 (2015).

**Faruque Ahmed**

USA

**Elie Akl**

Lebanon

**Waleed Alhazzani**

Canada

**Ahmed Ali**

Egypt

**Rivet Amico**

USA

**Gunnar Andersson**

USA

**Jorge Aparicio**

Spain

**Susan Armijo-Olivo**

Canada

**Garrett Ash**

USA

**Harold Atkins**

Canada

**Genesis Barbosa**

Brazil

**Anna Barrow**

UK

**Alicja Bartkowska-Sniatkowska**

Poland

**John Batchelor**

UK

**Suzanne Bench**

UK

**Nancy berkman**

USA

**Jesse Berlin**

USA

**Andrew Booth**

UK

**Alison Booth**

UK

**Lex Bouter**

Netherlands

**Fausto Bramante**

Brazil

**Sharon Brennan**

Australia

**Sue Brennan**

Australia

**Simon Briscoe**

UK

**Taylor Brown**

USA

**Felicity Brown**

USA

**Andrew Brown**

USA

**Susan Brunskill**

UK

**Ginny Brunton**

UK

**Deborah Caldwell**

UK

**Chris Cameron**

Canada

**Bridget Candy**

UK

**Valentina Cardi**

UK

**Kristin Carson**

Australia

**Adriana Castelli**

UK

**Ferrán Catalá-López**

Spain

**Michaël Chassé**

Canada

**Wei Chen**

China

**Hui-Ching Chuang**

Taiwan

**Katherine Clarke**

UK

**Shannon Cope**

Canada

**Alessandro Coppo**

Italy

**Daniel J Corsi**

Canada

**Trevor Cox**

UK

**Holger Cramer**

Germany

**Greta Cummings**

Canada

**Karina Davidson**

USA

**Thomas Debray**

Netherlands

**Cinzia Del Giovane**

Italy

**Jane Dennis**

UK

**Mary Dixon-Woods**

UK

**Jenny Doust**

Australia

**Therese Dowswell**

UK

**Simon Driver**

USA

**Preethy D'Souza**

Uruguay

**Sandy Dunn**

Canada

**Isabelle Durand-Zaleski**

France

**Solange Durao**

South Africa

**Sada Nand Dwivedi**

India

**Michele Dyson**

Canada

**Alison Eastwood**

UK

**Andem Effiong**

USA

**Doug Elliott**

Australia

**Julian Elliott**

Australia

**Alexandra Ellis**

USA

**Munira Essat**

UK

**Lise Estcourt**

UK

**Lise Jane Estcourt**

UK

**Blair Evans**

Canada

**Jonathan Evans**

UK

**Yngve Falck-Ytter**

USA

**Robin Featherstone**

Canada

**Deborah Finfgeld-Connett**

USA

**Padhraig Fleming**

UK

**Kate Flemming**

UK

**Jill Fortuin**

South Africa

**Geoff Frampton**

UK

**Thomas Fuller**

Netherlands

**Nicole Fusco**

USA

**Eric Galam**

France

**Chris Gale**

UK

**Feng Gao**

China

**Paul Garner**

UK

**Chantelle Garritty**

Canada

**Long Ge**

China

**Alessandra Gennari**

Italy

**Lise Gluud**

Denmark

**Su Golder**

UK

**Sean Grant**

USA

**Katja Grasic**

UK

**Richard Green**

UK

**Joanne Greenhalgh**

UK

**Vasudeva Guddattu**

USA

**Gouri Gupte**

USA

**Gordon Guyatt**

Canada

**Markus Haacker**

USA

**Zohar Habot-Wilner**

Israel

**David Hall**

UK

**Sonja Hansen**

Germany

**Rebecca Hardwick**

UK

**Tilahun Haregu**

Kenya

**James Hargreaves**

UK

**Sue Harnan**

UK

**Steve Harris**

UK

**Rebecca Hein**

Germany

**Julian Higgins**

UK

**Lena Holm**

Sweden

**Caroline Homer**

Australia

**T Itani**

USA

**Namiki Izumi**

Japan

**Justin Jagosh**

Canada

**Hayley Jones**

UK

**Jo Jordan**

UK

**Robert Kane**

USA

**Hannes Karin**

Belgium

**Monika Kastner**

Canada

**Cemil Kavalci**

Turkey

**Sara Khangura**

Canada

**Kamlesh Khunti**

UK

**Michal Kicinski**

Belgium

**Jos Kleijnen**

USA

**Evangelos Kontopantelis**

UK

**Daniel Korevaar**

Netherlands

**Felix Lambert**

UK

**Eddy Lang**

Canada

**Iain Lang**

UK

**Jose Lara**

UK

**Allana LeBlanc**

Canada

**Susan Lee**

USA

**Mariska MG Leeflang**

Netherlands

**Siu-Wai Leung**

Macao

**Melissa Glenda Lewis**

India

**James Lewis**

USA

**Kuancho Liao**

Taiwan

**Yu-Tsai Lin**

Taiwan

**Alexis Llewellyn**

UK

**Brynmor Lloyd-Evans**

UK

**Diana Lock**

USA

**Craig Lockwood**

Australia

**Christina Loitz**

Canada

**Li Lun**

China

**Vittoria Lutje**

UK

**Jinhui Ma**

Canada

**Steve MacGillivray**

UK

**Caroline Main**

UK

**Laxmaiah Manchikanti**

USA

**Iain Marshall**

UK

**Marrissa Martyn-St James**

UK

**Lynsay Matthews**

UK

**Evan Mayo-Wilson**

USA

**Catriona McDaid**

UK

**Jessie McGowan**

Canada

**Lauralyn McIntyre**

Canada

**Sionnadh McLean**

UK

**Susannah McLean**

UK

**Nick Meader**

UK

**Reint Meursinge Reynders**

Netherlands

**Louise Millard**

UK

**Wieneke Mokkink**

Netherlands

**Graham Moore**

UK

**Zachary Munn**

Australia

**Douglas Murphy**

UK

**Charles Murray**

UK

**Gillian Norman**

UK

**Gill Norman**

UK

**Chukwuemeka Nwachukwu**

Nigeria

**Catharina Nygren deBoussard**

Sweden

**Eleanor Ochodo**

Netherlands

**Karen Oechsle**

Germany

**Martin Offringa**

Canada

**Jonathan Olsen**

UK

**Dervla O'Malley**

Ireland

**Maya O'Neil**

USA

**E Otto-Buczkowska**

Poland

**Neville Owen**

Australia

**Matthew Page**

Australia

**Kingshuk Pal**

UK

**Raul Palacio-Rodriguez**

UK

**Shu Ming Pan**

China

**Nikolaos Pandis**

USA

**Anand Pandyan**

UK

**Kiran Patel**

UK

**Porjai Pattanittum**

Thailand

**Alan Pearce**

Australia

**Mark Pearson**

UK

**Tatiana Pereira-Cenci**

Brazil

**Kai Petersen**

Sweden

**Martine Piccart**

Belgium

**Dawid Pieper**

Germany

**Josiah Poon**

Australia

**Jennie Popay**

UK

**Jerilynn Prior**

Canada

**Rebecca Rees**

UK

**Christoph Reiners**

Germany

**Bernd Richter**

Germany

**David Riley**

USA

**Shannon Robalino**

UK

**Helen Roberts**

UK

**Bram Rochwerg**

Canada

**Mark Rodgers**

UK

**Anke Rohwer**

South Africa

**Marta Roque**

Spain

**Gerta Rücker**

Germany

**Peter-Michael Sack**

Germany

**Ian Saldanha**

USA

**Margaret Sampson**

Canada

**Alvaro Sanabria**

Colombia

**Nancy Santesso**

Canada

**Jelena Savovic**

UK

**AB Schneider**

USA

**Vanadin Seifert-Klauss**

Germany

**Shelley Selph**

USA

**Soman Sen**

USA

**Larissa Shamseer**

Canada

**Aryeh Shander**

USA

**Paul Shekelle**

USA

**Jonathan Shepherd**

UK

**Mark Simmonds**

UK

**E Simpson**

UK

**Becky Skidmore**

Canada

**Birte Snilstveit**

UK

**Karla Soares-Weiser**

UK

**Robabeh Soleimani**

Iran

**Harriet Sommer**

Germany

**Jayadevan Sreedharan**

India

**J Bart Staal**

Netherlands

**Claire Stansfield**

UK

**Rebecca States**

USA

**John Steinke**

USA

**Adrienne Stevens**

Canada

**Sharon Straus**

Canada

**Carolyn Summerbell**

UK

**Andrzej Szopa**

Poland

**Siriwan Tangjitgamol**

Thailand

**Emily Tanner-Smith**

USA

**Nancy Temkin**

USA

**Caroline Terwee**

Netherlands

**Kednapa Thavorn**

Canada

**Sudhin Thayyil**

UK

**James Thomas**

UK

**Brett Thombs**

Canada

**Jennifer Thorne**

USA

**Stephanie Tierney**

UK

**Adrian Tookman**

UK

**Francine Toye**

UK

**Adrian Traeger**

Australia

**Alexander Tsertsvadze**

Canada

**Lorainne Tudor Car**

UK

**Lucy Turner**

Canada

**Claire Twose**

USA

**Antonis Valachis**

Sweden

**Peter Van Muijen**

Netherlands

**Gert van Valkenhoef**

Netherlands

**Arthur van Zanten**

Netherlands

**Veljko Veljkovic**

Russian Federation

**Jos Verbeek**

Finland

**Areti Angeliki Veroniki**

Canada

**Varsha Vimalananda**

USA

**Jean-Louis Vincent**

Belgium

**Byron Wallace**

Usa

**Tanya Walsh**

UK

**Marta Wanat**

UK

**Qi Wang**

China

**Luhua Wang**

China

**Hugh Watson**

France

**Alison Weightman**

UK

**Emma Welsh**

UK

**Maria M. Wertli**

Switzerland

**Kim Wever**

Netherlands

**Evelyn Whitlock**

USA

**Alexis Whitton**

Australia

**Paul M Wilson**

UK

**Evelyn Wong**

Australia

**Geoff Wong**

UK

**Nerys Woolacott**

UK

**Judy Wright**

UK

**Kath Wright**

UK

**Changchun Xie**

USA

**Li Xiuxia**

China

**Kehu Yang**

China

**Yue Yang**

China

**Flora Zagouri**

Greece

